# Measuring social exclusion and its distribution in England

**DOI:** 10.1007/s00127-023-02489-x

**Published:** 2023-05-09

**Authors:** Jennifer Dykxhoorn, David Osborn, Laura Fischer, David Troy, James B. Kirkbride, Kate Walters

**Affiliations:** 1 Department of Primary Care and Population Health, UCL, Rowland Hill Street, London, NW3 2PF UK; 2 Division of Psychiatry, UCL, 6th Floor, Maple House, 149 Tottenham Court Road, London, W1T 7NF UK; 3 Camden & Islington NHS Trust, 4 St Pancras Way, London, NW1 0PE UK; 4 Bristol Population Health Sciences Institute, University of Bristol, Canynge Hall, Bristol, BS8 2PS UK

**Keywords:** Social exclusion, Public health, Principal component analysis, Understanding Society, Health inequalities

## Abstract

**Background:**

Social exclusion is a multidimensional concept referring processes which restrict the ability of individuals or groups to participate fully in society. While social exclusion has been used to explore patterns of disadvantage, it has been difficult to measure. Thus, we aimed to use population-based data to measure social exclusion and its constituent domains and to describe its distribution in England.

**Methods:**

We used data from Understanding Society in 2009/2010 develop a multidimensional measurement approach, replicated in 2018/2019. We defined five domains of social exclusion from the literature and expert consultation: material, relational, political, digital, and structural. In both waves, we identified measures for each domain, then conducted principal component analysis to identify the components. We generated domain scores and an overall social exclusion score. We described the distribution of social exclusion and its domains by sex, region, age, and ethnicity.

**Results:**

We found the level of social exclusion was higher in the youngest age group and decreased by age. We found elevated levels of overall social exclusion for ethnic minoritised groups including African, Arab, and Caribbean groups compared to White British groups. We found distinct patterns within each domain.

**Discussion:**

We developed an overall measure of social exclusion with five domains, and finding distinct patterns of social exclusion by age, ethnicity, and region which varied across domain. These findings suggest that attention should be paid to the separate domains due to different population distributions. This measurement approach moves beyond conceptual discussions of social exclusion and demonstrates the utility of a quantitative measure of social exclusion for use in health and social research.

**Supplementary Information:**

The online version contains supplementary material available at 10.1007/s00127-023-02489-x.

## Introduction

The term social exclusion first emerged in the 1970s, and in the intervening decades, it has had a central role in policy discourse in the UK and Europe. Despite this long history, social exclusion is a conceptually complex term, with its meaning continues to change and evolve depending on context and political ideology [1]. The common aspects across the three dominant paradigms of social inclusion are the process orientation which integrates poverty, employment, social integration, and social justice in a multidimensional concept [2]. While social exclusion has an intuitive appeal, the lack of clarity around its definition has hampered its integration into analytic work.

Social exclusion is a multidimensional concept which refers to processes which restrict groups or individuals from participating fully in society [3, 4]. Social exclusion is linked to many related concepts, including poverty, deprivation, unemployment, and low social capital. Further, it is a relational process by which power hierarchies result in the unequal distribution of resources [3, 5]. Unequal power structures are a central concept in the monopoly paradigm of social exclusion, proposed by Silver (1995) and used extensively in the European policy literature [1]. This paradigm posits that the social exclusion conceptually encompasses broader power structures and decision-making at the community, institutional, and structural level which serve the interests of the included and result in the systematic exclusion of certain groups of people from opportunities [1, 6, 7]. This concept represents a system-based understanding of how the broader social structure creates and recreates conditions to advantage or disadvantage certain groups. Social exclusion, therefore, can be used to describe patterns of disadvantage and has the potential to shift focus to social justice and greater equity making it a key concept for public health research and action [3].

Public health has an important role to play in advocating for socially excluded groups and adapting policies in order to meet the needs of these groups [4]. Social exclusion has been on the political agenda with governmental bodies established to investigate social inclusion and exclusion [8, 9]. In the United Kingdom (UK), the Social Exclusion Task Force was established in 1997 to guide the government’s response to social exclusion [10]. Social exclusion has been identified as a potentially important tool for understanding mental health problems as there is a bi-directional relationship between social exclusion and mental health problems. People who experience mental health problems are at increased risk of experiencing social exclusion [11, 12], and simultaneously, the experience aspects of social exclusion have been shown to increase the risk of mental health problems [13, 14]. While social exclusion may be a useful conceptual tool for public mental health policy and practice [15, 16], its utility is limited without a consistent definition and clear measurement approaches.

While there remains some debate on the precise definition of social exclusion, several have attempted to measure it analytically [17–20]. Previous quantitative research has used a single measure as a proxy for social exclusion, focussing on poverty or voting. While these represent aspects of social exclusion, single measures are not able to capture the ways that people constrained in their ability to participate economically, socially, and politically in society. In order for social exclusion to be a useful tool for public health, we need to be able to measure it accurately [14, 21, 22].

Several studies have been conducted with the explicit goal of measuring social exclusion, including the UK’s Millennium Survey of Poverty [23] and Social Exclusion and the Australian Community Understanding of Poverty and Social Exclusion Survey [8]. These surveys provided a detailed snapshot of social exclusion; however, due to their cross-sectional nature and small sample size, they were not able to describe the extent of exclusion in population subgroups and over time. In 2017, Dutch researchers published a Social Exclusion Index for Health Surveys (SEI-HS) [24] with four domains: material deprivation, limited social participation, inadequate access to basic social rights, and lack of normative integration. While this index represents an advancement in measuring social exclusion, it contains measures that are not routinely collected in other countries, including the UK.

The primary objective of this study was to define the key domains of social exclusion and develop a measurement approach estimate social exclusion and its constituent domains using data from a longitudinal survey in England. The secondary objective of this study was to use this measurement approach to describe the distribution of social exclusion in England across two survey waves.

## Methods

### Data

We used data from Understanding Society (USoc), a longitudinal household study from the UK [25]. USoc is a nationally representative household panel survey designed using stratified, clustered equal probability sample design [26]. All household members ages 16 or older of a selected household are interviewed annually. Most interviews occur face-to-face in the respondents’ home by trained interviewers or by the respondents online, while some participants complete the survey by telephone [26]. In Wave 1, the questionnaires were available in ten languages: Arabic, Bengali, Cantonese, English, Gujarati, Punjabi Gurmukhi, Punjabi Urdu, Somali, Urdu, and Welsh, and translated interviews were done using Unicode Translation Interview Programme [27]. Additional translation would be arranged if the participant could not speak English, or one of the nine translations prepared for USoc data collection [27]. In Wave 1, 57.1% of eligible households responded, which included 40,000 household responses.

### Sample

We included adults aged 16 or older (*n* = 31,100) who were living in England in Wave 1 (2009/2010) and Wave 10 (2018/2019; *n* = 25,559). We excluded proxy respondents and fed-forward values from Wave 9 (2017/2018) for measures which were not included in Wave 10.

### Exposure

We reviewed Medline, Cochrane Library, and Web of Science for definitions and measures of social exclusion (Supplement A). Six potential domains were identified and presented to an expert advisory panel of academics, public health practitioners, clinicians, and members of the public. These domains were then re-organised into five domains: material, relational, political, digital, and structural (Table 1).

**Table 1:** Social exclusion domains and definitions

Domain	Definition
Material exclusion	Material exclusion includes the lack of resources to support basic living conditions, including economic strain (e.g. low income, difficulty affording food, housing, or unexpected expenses), inadequate housing conditions (e.g. damp, rot, inadequate heating), and enforced lack of consumer durable items (e.g. items that people would like to possess but cannot afford them including telephone, car, washing machine) [40]. Measures of material exclusion are often measured at the individual or household level but can also include area-based and collective resources
Relational exclusion	Relational exclusion includes lack of meaningful social relationships, including presence and satisfaction with close relationships, experiences of loneliness and isolation, relationships to those in the local area, and ties to a broader social network. Relational exclusion can be measured by frequency of contact, closeness, or satisfaction of various relationships and sense of belonging to a social group
Political exclusion	Political exclusion includes not being able to fully participate in the political process, taking collective action, or participating in local, regional, or national decision making. Political exclusion also includes barriers to participating in protests, awareness-raising, or grass-roots activism to affect political decisions or make change
Digital exclusion	Digital exclusion includes barriers to accessing digitally-available information and services due to lack of technology (e.g. computers, internet connection, or mobile devices) or low levels of digital literacy and computer skills [36]
Structural exclusion	Structural exclusion describes those who are excluded due to the organisation of society which dictates who has access to power, who is afforded human rights and social justice, and who is subjected to negative cultural assumptions and discrimination. Structural exclusion can also include how the distribution of the wealth creates inequalities, intergenerational transmission of advantage and disadvantage, and how private companies and media contribute to the creation of advantaged and disadvantaged positions in society

### Item selection

We used survey items in Wave 1 to identify variables for each domain. To measure material exclusion, we included 11 measures of income, employment, and housing (Supplement B).

We included six measures of relational exclusion: household composition, marital status, relationship satisfaction + cohesion, contact with family, importance of local friendships, and neighbourhood cohesion. In Wave 10, we used loneliness and  social isolation instead of relationship satisfaction and cohesion.

Political exclusion was measured using seven measures: supports a political party, level of interest in politics, satisfaction with environmental habits, environmental lifestyle, belief that individual action can contribute to climate change, belief that it is worth taking individual action, participates in community groups, and attends religious services.

Digital exclusion was estimated using information provided by the primary household respondent, including access to a computer and internet at home and access to a mobile phone. We included frequency of internet use in the Wave 10.

We included four measures of structural exclusion: difficulty with day-to-day English, intergenerational educational mobility, intergenerational occupational mobility, and regional wealth inequality.

### Statistical analysis

#### Principal component analysis

To reduce the data’s dimensionality while preserving variability [28], we conducted Principal Component Analysis (PCA) for each domain in Wave 1, which we repeated in Wave 10. Within each wave, we included exposure variables within each domain. We used the Kaiser–Meyer–Olkin (KMO) measure of sampling adequacy to verify that there was enough correlation in the variables within each domain to warrant a PCA approach, using the cutoff of 0.5 or more [29]. We used a pre-defined cut-point off to retain sufficient components to explain 65% or more of total variability in each domain [28]. We inspected the eigenvalues to ensure they met the Kaiser criterion of being greater than 1. When the criterion disagreed, we selected the number of factors that met the variability cutoff. To maximise the correlations on the fewest components, we used oblique rotation (promax). We created standardised scores for each domain based on the selected number of components, with a mean of 0 and standard deviation of 1, where negative scores indicated lower levels of exclusion and positive scores indicated higher levels of exclusion. These standardised domain scores were then summed to create an overall social exclusion score, allowing each domain to have equal weight in the overall score. We conducted a PCA analysis on all exposure variables to assess if the measures loaded into the similar domains and components (Supplement B).

#### Descriptive analysis

We explored the distribution of the social exclusion score and domain-specific scores by sex, region, age, and ethnicity. Analysis was conducted using Stata (version 17) and regional visualisation was conducted in ArcGIS (version 10.8). We used the same colour scheme across all tables and figures, using darkening green to indicate higher exclusion scores and darkening purple to indicate lower exclusion scores, with scores around the mean (− 0.4 to 0.4) left white. The study protocol was published in October 2020 (https://osf.io/ag8ec/).

## Results

### Sample characteristics (Wave 1)

There were 31,100 individuals included in our Wave 1 analysis, including more females than males (55.6% and 44.4%, respectively). 77.9% of participants had a White British ethnicity, and there were participants from 12 other ethnic backgrounds. The proportion of participants from each region ranged from 5.0% in the North East to 16.0% in London.

We conducted Principal Component Analysis (PCA) within each domain in Wave 1.

### Material exclusion

We included 11 measures of material exclusion, which had a KMO measure of sampling adequacy of 0.77. Inspection of the eigenvalues indicated that only three components had exceeded the eigenvalue of 1; however, in keeping with our a priori criterion, five components were retained to achieve our pre-defined cutoff of explaining at least 65% of the total variance (Table 2; Supplement C). Component 1 (income) included subjective financial status, income satisfaction, and ability to afford material goods and pay bills. Component 2 (employment) included employment status and job satisfaction. Component 3 (housing) included housing affordability and tenure. Component 4 included educational attainment and income fifths, and Component 5 included desire to move house, with strong loading by the variable “if you could choose, would you stay here in your present home or would you prefer to move somewhere else.”

**Table 2:** Social exclusion domains and constituent components

Domains	Material exclusion	Relational exclusion	Political exclusion	Digital exclusion	Structural exclusion
Components	M1. Income	R1. Household relationships	P1. Political interest	D1. Household technology and connectivity	S1. Intergenerational mobility
	M2. Employment	R2. Extended family contact	P2. Worth taking individual action		S2. Difficulty with day-to-day English
	M3. Housing	R3. Neighbourhood relationships	P3. Attending services and taking action		S3. Regional wealth inequality
	M4. Education		P4. Environmental lifestyle		
	M5. Desire to move house				

### Relational exclusion

Six variables were included in the relational exclusion analysis with an overall KMO of 0.50. We retained three components which had eigenvalue over 1 and explained 66.9% of the variance (Table 2). Component 1 (household relationships) included household composition, marital status, and relationship satisfaction + cohesion. Component 2 included frequency of contact with extended family, and Component 3 included local friendships and neighbourhood cohesion.

### Political exclusion

There were seven measures of political exclusion with an overall KMO of 0.54. Four components had eigenvalues over 1, explaining 67.5% of the variance (Table 2). Component 1 (political interest) included support for a political party and level of interest in politics. The belief that it is worth taking individual action, even if others are not doing the same was the only variable which loaded on Component 2. Component 3 included attending religious services and taking individual action and Component 4 was environmental lifestyle and satisfaction with environmental habits.

### Digital exclusion

The two variables included in the digital exclusion domain had an overall KMO of 0.50. We retained one component (technology and connectivity), as it had an eigenvalue of 1.36 and explained 67.8% of the variance (Table 2).

### Structural exclusion

The structural exclusion domain was comprised of 4 variables and the overall KMO was 0.52. While only two components had eigenvalues over 1, we retained 3 components to explain > 65% of the variance (Table 2). Both measures of intergenerational mobility loaded strongly onto Component 1, difficulty with day-to-day English loaded onto Component 2, and the final component was regional wealth inequality.

### Overall PCA

The overall PCA showed that variables broadly clustered according to the five domains and followed similar component loading (Supplement C).

### Calculation of scores

We generated standardised domain scores with the mean of 0 and standard deviation of 1 using the PCA components. We calculated an overall social exclusion score by summing domain scores.

### Sample characteristics (Wave 10)

There were 25,559 participants in Wave 10 with a similar sex distribution compared to Wave 1 (55.4% females, 44.6% males). The largest ethnic group continued to be White British, but this had decreased slightly to 74.4%. The North East had the smallest proportion (4.4%) with London continuing to have the largest proportion of participants (15.1%). We repeated the above approach to variables in Wave 10 (Supplement C).

### Descriptive results

#### Wave 1

**Fig. 1:**
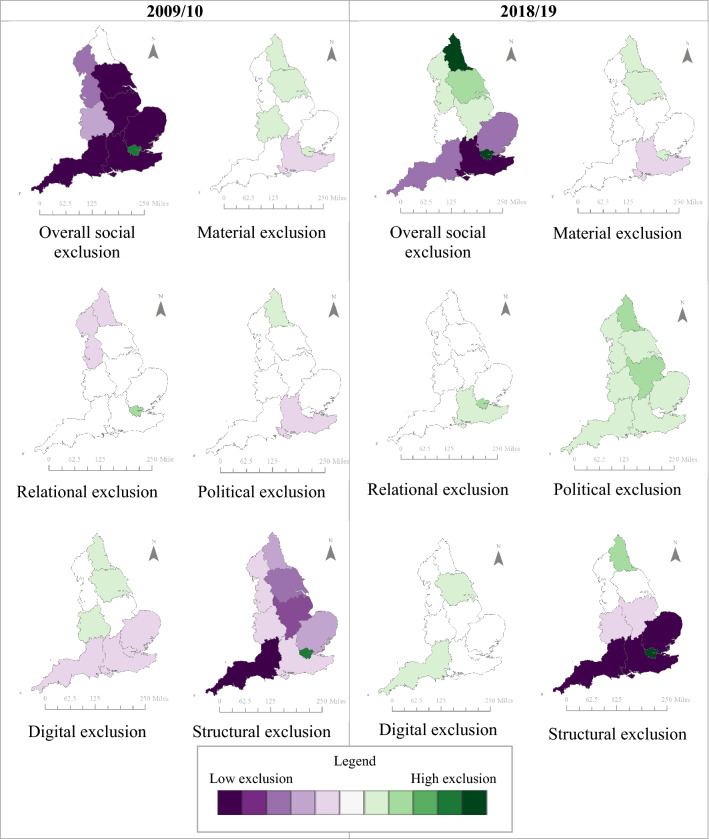
Social exclusion domains by region, 2009/2010 and 2018/2019

**Fig. 2:**
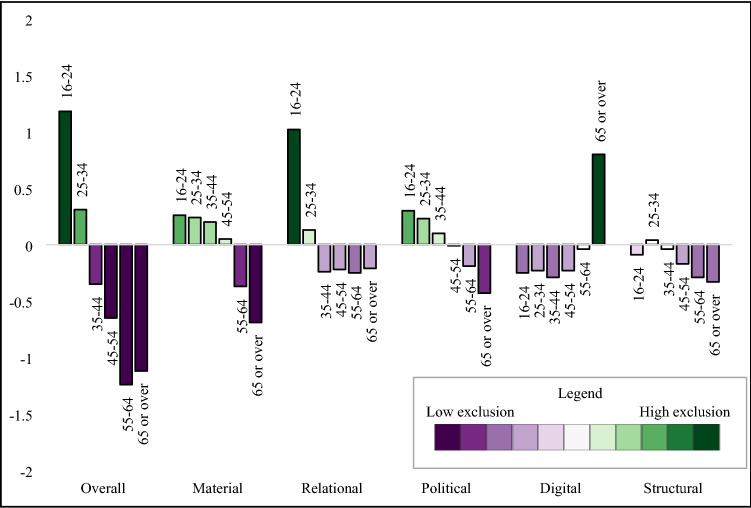
Social exclusion domains by age group, 2009/2010

**Fig. 3:**
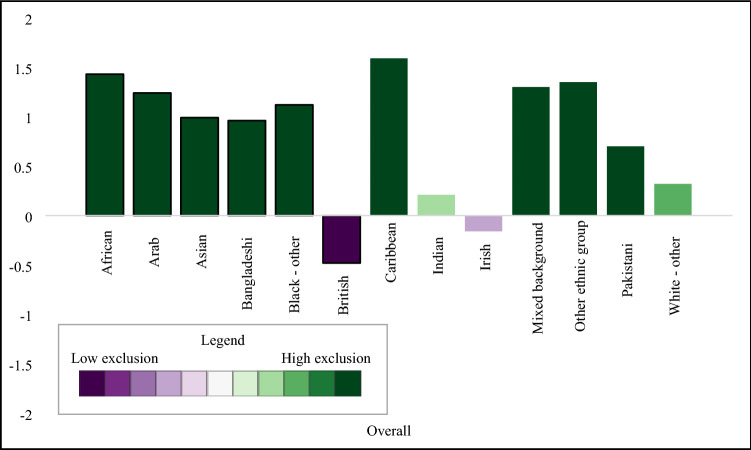
Overall social exclusion by ethnicity, 2009/2010

We found negligible differences of social exclusion by sex, with males having higher levels of relational exclusion and females with higher levels of political and digital exclusion (Table 3). London had the highest levels of overall social exclusion, including elevated scores in relational and structural domains (Fig. 1). The South West region had the lowest levels of overall exclusion, with low levels of exclusion across all domains. The regional patterns of overall social exclusion appeared to be driven by differences in the structural domain. We observed a linear decrease in overall social exclusion by age group (Fig. 2) with high levels in the youngest age groups, and lower levels in the oldest age groups. Specifically, 16–24-year-olds and 25–34-year-olds had elevated levels of material, relational, and political exclusion, and political exclusion, while those 65 or older had low levels of social exclusion in all domains except digital. We found large differences by ethnicity, with high levels of overall exclusion in high levels in African, Caribbean, Arab, Bangladeshi, Black (other), Asian, mixed, other ethnic backgrounds compared to low social exclusion in the White British group (Fig. 3).

**Table 3:**
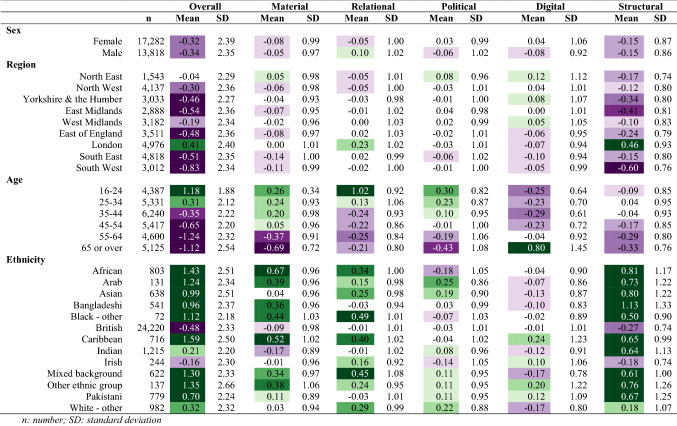
Social exclusion observations, weighted mean, and standard deviation in 2009/2010, *n* = 31,100

#### Wave 10

The distribution of social exclusion in Wave 10 was similar to that in Wave 1 by sex, age, and ethnicity, but there were notable differences by region. While high levels of social exclusion in London persisted, social exclusion was also elevated across all northern regions (Fig. 1). The South East, South West, and East of England had the lowest levels of overall social exclusion, driven by low levels of structural exclusion.

**Fig. 4:**
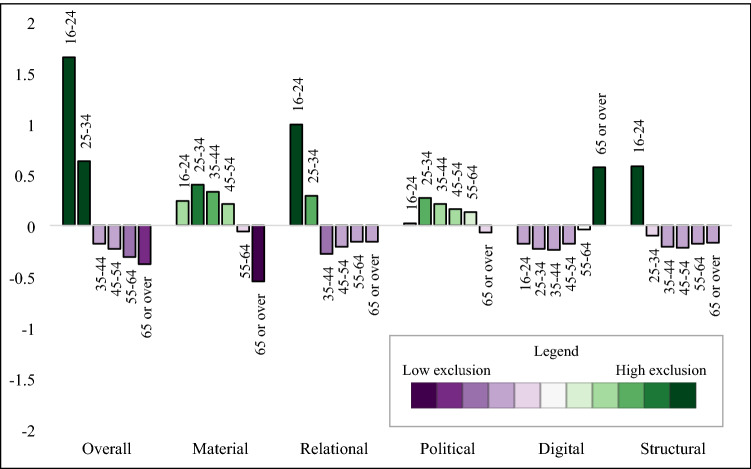
Social exclusion domains by age group, 2018/2019

Across the domains of social exclusion, there was stability in the material and relational domains by age and ethnicity. In the political domain, we observed an increase of political exclusion in every region, with the North East and the East Midlands showing the largest increase in political exclusion. We also noted a large reduction in political exclusion in the youngest age group in 2018/2019 compared to 2009/2010 (Table 4; Fig. 4). There were several shifts in the distribution of digital exclusion, with increased digital exclusion noted in the Irish and Black (other) groups (Table 4). Further, patterns of digital exclusion by region were largely eliminated by 2018/2019, with the exception of slightly elevated rates in Yorkshire and the Humber and the South West (Fig. 1). The level of digital exclusion in the oldest age group was still higher than other age groups but had somewhat attenuated by Wave 10 (Fig. 4).

**Table 4:**
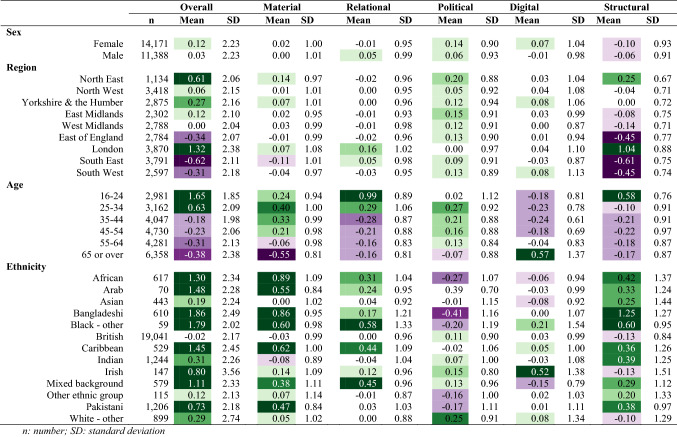
Social exclusion observations, weighted mean, and standard deviation in 2018/2019, *n* = 25,559

## Discussion

**Fig. 5:**
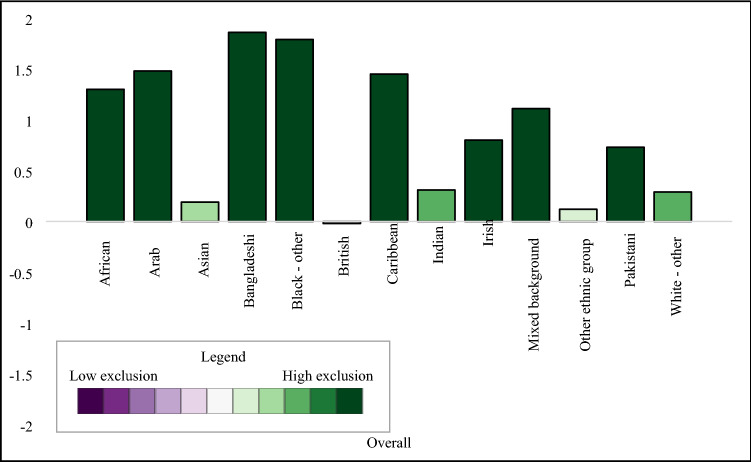
Overall social exclusion by ethnicity, 2018/2019

Social exclusion has been discussed in many disciplines for decades, yet previous efforts to measure this exclusion have not captured the multidimensional complexities of social exclusion. We developed a five-domain model of social exclusion: material, relational, political, digital, and structural, based on the academic literature and expert consultation. We used items from a longitudinal household survey to parameterise this model, creating domain-specific and overall social exclusion scores (Fig. 5).

We showed how social exclusion varied by age, ethnicity, and region, with high levels of social exclusion experienced in the youngest age groups, minoritised ethnic groups, and those living in London or the north of England. We found striking differences by domain. Taken together, these results demonstrate the utility of a multidimensional measure to provide overall and domain-specific estimates of social exclusion.

When comparing the estimates from 2009/2010 to 2018/2019, we found stability for social exclusion and its domains by age, showing decreasing levels of social exclusion with increasing age. Further, levels of social exclusion were higher in ethnically minoritised groups compared to the White British group at both time points, which appeared to be driven by material, relational, and structural exclusion. We noted interesting changes by geographic region between the two time points. In 2009/2010, four regions had very low levels of social exclusion: South West, East Midlands, South East, and Yorkshire and the Humber. In contrast, the results from 2018/2019 showed clearer evidence of a North–South divide in exclusion scores, where regions north of the Severn-Wash line had high levels of social exclusion (North East, Yorkshire and the Humber, North West, East Midlands), while Southern regions had low levels. While this may indicate growing regional disparities in social exclusion, more work is needed to explore the sensitivity of this measurement approach and its stability over time.

There is some debate in the literature about if someone would be considered socially excluded if they experience exclusion on a single domain. Some research suggests that individuals must face exclusion in multiple domains to be considered excluded, while others maintain that low participation and exclusion in any domain is sufficient to being classified as excluded [10, 30]. In our measurement approach, we used standardised sums for each domain to create an overall measure of social exclusion, describing those with scores above the mean as experiencing social exclusion, to varying degrees. However, as this is a mean score based on domain scores, groups may experience high levels of exclusion in one domain and low levels of exclusion in another, so would receive a social exclusion score near the mean. Rather than focussing on a cutoff point, we suggest that these measures should be viewed as a continuum, which allowed a more nuanced understanding of social exclusion. For example, an individual may experience high exclusion in a single domain, while others may experience moderate levels of exclusion across multiple domains. These differing circumstances would require different policy and intervention responses.

While it is useful to have an overall measure of social exclusion, the summary score masks the heterogeneity in the domains. Being able to disaggregate social exclusion into its parts can be useful to inform public health interventions. For example, while those 65 or older had the lowest overall social exclusion scores at both time points, the high level of digital exclusion indicates that this group may benefit from interventions with support their digital inclusion. The youngest age group had the highest level of social exclusion across the age groups, which was driven by high levels of relational exclusion and elevated scores in the structural and material domains. This trans-domain elevation of social exclusion may require a multi-faceted public health intervention which supports young people in developing positive social relationships, supports their inclusion in employment and access to adequate housing, and addresses structural barriers. This research highlights different drivers of social exclusion in population groups. This may inform targeted public health responses to reducing exclusion and supporting inclusion of vulnerable population groups.

### Comparison to previous literature

The five domains in our social exclusion score build on previous work measuring social exclusion, most notably the Social Exclusion Index for Health Surveys (SEI-HS) [24]. SEI-HS’s material deprivation domain was like our material exclusion domain, the social participation domain was analogous to our relational exclusion domain, and measures within “lack of normative integration” were like measures in our political exclusion domain. We expanded political exclusion to include political participation and engagement in addition to contributing social causes and acting for collective good. We included digital and structural exclusion, which were not measured in the SEI-HS, but were supported by our literature review of social exclusion and consultations with our expert advisory panel. While there was overlap in our measurement approaches, the SEI-HS was derived based on measures in the Netherlands Public Health Monitor, which included many items which are not routinely collected in the UK, including feeling cutoff from people, feeling rejected, having people who understand you, receipt of medical and dental treatment, and giving to good causes. Future research could investigate how the SEI-HS compares to our measurement approach if a suitable dataset is identified. USoc is part of a world-wide group of household panel surveys set up to allow cross-national comparisons. This harmonisation project aims to make the data collected in USoc comparable with long-running studies in other countries, including Australia, Germany, Korea, Switzerland, and the United States [31]. Due to this alignment of variables, future studies may be able to replicate this measurement approach in other contexts.

The summary measure of social exclusion was able to highlight patterns across key demographic factors and our findings align with previous literature. The overall score, for example, provided evidence of the North–South divide, a geographic phenomenon observed in previous research where regions north of the Severn-Wash line experience higher levels of deprivation, higher levels of premature mortality, and other poor health outcomes compared to than southern regions [32, 33]. With the notable exception of London, southern regions had lower social exclusion than the rest of England. These differences were particularly apparent in the material and structural domains. The high level of social exclusion in London aligned with previous findings in older adults which showed that there was elevated social exclusion across multiple domains [22]. In contrast to previous findings [34, 35], we found a strong inverse relationship between age and social exclusion, with greater social exclusion at younger ages.

### Limitations

While USoc includes many measures, the available variables did not fully capture the theoretical concepts for each domain. For example, there were few variables available for the digital domain and were not adequate to capture variations in access, resources, knowledge, and skills. In 2018/2019, most households reported having a computer and internet connection, but the persistent digital divide in England suggests that access alone is not sufficient for digital inclusion [36, 37]. An important construct within the structural domain was discrimination; however, discrimination questions were only asked of less than 5% of the sample so had to be excluded. In the political domain, we wanted to estimate taking collective action but were limited to using environmental habits as a proxy, which misses other ways an individual may take action. Further, while we acknowledge that social exclusion is shaped by broad structural factors, including social and cultural norms, government policies, wider economic conditions, and global events [38, 39], these were not captured in USoc and our analysis was conducted primarily on individual- and household-level variables. More broadly, there are some differences in demographics between study participants and the UK population. For example, there is a lower proportion of participants born outside of the UK in the study (12.6%) compared to the UK average (16.8%). There may be other differences which limit the generalisability of this study to the UK. We recommend weighting estimates of social exclusion in studies applying this measurement approach in order to address demographic differences between USoc participants and the general population.

This research focussed on the experience of social exclusion which was non-voluntary and linked to broader circumstances and systems which prevented full participation in society which were beyond the control of an individual. Some individuals choose not to participate in aspects of society, like choosing not to be involved in political processes or choosing to eschew technology due to personal beliefs and preferences. We were not able to determine if participants chose to not participate in various aspects of life (voluntary) or if they were socially excluded (non-voluntary).

### Strengths

Despite limitations in the measures, USoc includes extensive measures which made it possible to explore social exclusion in detail, which might not be possible in other studies. This approach captures the multidimensional nature of social exclusion. Our domains overlap with and extend previous research, by adding a digital exclusion domain, which has not appeared in previous indices and has emerged as an increasingly relevant aspect of exclusion.

A further strength of this approach is that it outlines a method which can be used to generate comparable social exclusion scores which can be used in population surveys, even if the exact measures are not included. This measurement approach may improve the generalisability of this measurement approach, as future waves of this survey and other population surveys may use different measures of income or education, so by including the available measures which best estimate the constituent components of each domain, we would expect broadly similar results. This represents a pragmatic use of existing data, as population surveys have been developed to capture a broad picture of the health of the population and often do not have social exclusion as their primary focus. This allows us to estimate social exclusion using existing data. The flexible and pragmatic approach to estimating the domains permits longitudinal follow-up in the context of changing survey design. It also allows for the estimation of regional levels of social exclusion, which was not possible in smaller surveys.

### Conclusions

We generated overall social exclusion scores and five domain scores to measure social exclusion. The persistently high level of social exclusion experienced by minoritised ethnic  groups and the youngest age groups over time highlighted a lack of progress in reducing inequalities over time.

This approach to measuring social exclusion demonstrates the utility of population-based surveys to estimate multidimensional concepts. Using multiple measures and principal component analysis, we developed a flexible measurement approach, which may increase the utility of this approach in population health research where identical measures are not available. While further research is needed to validate this measurement approach in other datasets, this research demonstrates the utility of estimating social exclusion in population surveys to answer questions of public health import.

### Supplementary Information

Below is the link to the electronic supplementary material.

Supplementary file1 (DOCX 112 KB)

## Data Availability

Understanding Society data are available through the UK Data Service. Researchers who would like to use Understanding Society need to register with the UK Data Service before being allowed to apply for and download datasets.
