# Supplementary material for: Measuring social exclusion and its distribution in England
Source: Soc Psychiatry Psychiatr Epidemiol. 2023 May 9;59(1):187–98. doi: 10.1007/s00127-023-02489-x (PMC10799797; doi:10.1007/s00127-023-02489-x)
Supplement: Supplementary file 1 — Supplementary file1 (DOCX 112 KB) [file 127_2023_2489_MOESM1_ESM.docx]

**Supplement A: Defining the domains of social exclusion**

We searched Medline, the Cochrane Library, and Web of Science for definitions and measures of social exclusion published using the search terms (title/ abstract/ keyword) “social exclusion” OR “social exclus*”. The initial search was conducted in February 2020, and results were presented to an expert advisory group, comprised of academic researchers, public health practitioners, policy makers, clinicians, and experts by lived experience to determine the domains within social exclusion. Preliminary domains were (1) Material deprivation, (2) Social interaction, (3) Participation in decision making & access to power, (4) Access to public and private services, (5) Structural issues, (6) Cultural / social position/ identity. Advisory panel included academic experts, practitioners, and members of the public met on 1 April 2020. Due to overlap between *Participation in decision making and access to power* and *Access to public and private services* the advisory group decided these would be best combined into a single domain. Additionally, the final domain, including gender/sex, ethnicity, religion, and other aspects of identity was thought to be the key demographics that the measures of social exclusion should be summarised for, not an independent domain. In other words, these are factors that affect the ability to fully participate in each domain. During a second advisory panel meeting (September 2021), it was decided that variables related to digital exclusion, which had previously been included within the material and political domains, should be separated into a distinct digital domain, and the search was updated (September 2021).

**Supplemental table 1 Selected definitions and domains of social exclusion in previous studies**

| **AUTHOR** | **YEAR** | **DEFINITION** | **DOMAINS** |
| --- | --- | --- | --- |
| Barnes et al. ^22^ | 2006 | Social exclusion refers to the multidimensional and dynamic process of being shut out, fully or partially, from the economic, social, and cultural systems that determine the social integration of a person in society (pg. 15) | 1. Exclusion from social relationships 2. Exclusion from cultural activities 3. Exclusion from civic activities 4. Exclusion from access to basic services 5. Neighbourhood exclusion 6. Exclusion from financial products 7. Exclusion from material goods |
| Burchardt et al. ^8^ | 1999 | An individual is socially excluded if (a) he or she is geographically resident in a society and (b) he or she does not participate in the normal activities of citizens in that society (pg. 229). | 1. Consumption activity 2. Savings activity 3. Production activity 4. Political activity 5. Social activity |
| Byrne | 1999 | Social exclusion is defined as a multidimensional process, in which various forms of exclusion are combined. When combined, they create acute forms of exclusion that find a spatial manifestation in particular neighbourhoods. | 1. Participation in decision-making and political processes 2. Access to employment and material resources 3. Integration into common cultural practices |
| Commins ^28^ | 1993 | Social exclusion defined as a failure in one or more of the four domains (civic, economic, social, or interpersonal). | 1. Democratic and legal for civic integration 2. Labour market for economic integration 3. Welfare state for social integration 4. Family and community for interpersonal integration |
| de Haan ^19^ | 1999; 2001 | “[S]ocial exclusion is a theoretical concept, a lens through which people look at reality and not reality itself (pg. 28).” | 1. Economic 2. Political 3. Social |
| Estivill ^29^ | 2003 | “Social exclusion may therefore be understood as an accumulation of confluent processes with successive ruptures arising from the heart of the economy, politics and society, which gradually distances and places persons, groups, communities and territories in a position of inferiority in relation to centres of power, resources and prevailing values.” (pg. 19) |  |
| Eurostat ^30^ | 2010 | “Social exclusion relates to being unable to enjoy levels of participation that most of society take for granted. It is a complex, multidimensional, and dynamic concept.” (pg. 11) | 1. Labour market exclusion 2. Education-related exclusion 3. Health-related exclusion 4. Housing-related exclusion 5. Exclusion from social networks and the information society |
| Fleury ^19^ | 1998 | Exclusion is a cultural process that implies the establishment of a norm that prohibits the inclusion of individuals, groups, and populations in a socio-political community.  (pg. 13) | 1. Economic 2. Political 3. Social |
| Gordon *et al*., ^13^ | 2000 |  | 1. Impoverishment (exclusion from adequate income or resources) 2. Labour market exclusion (exclusion from paid work) 3. Service exclusion (exclusion from public and private services) 4. Exclusion from social relations |
| Levitas et al., ^9^ | 2007 | ﻿Social exclusion is a complex and multidimensional process. It involves the lack or denial of resources, rights, goods and services, and the inability to participate in the normal relationships and activities, available to the majority of people in a society, whether in economic, social, cultural, or political arenas. It affects both the quality of life of individuals and the equity and cohesion of society as a whole (pg. 25). | 1. Resources (material and economic resources, access to services, social resources) 2. Participation (economic social, cultural, political, civic participation) 3. Quality of life (health and wellbeing, living environment, harm, and criminalisation) |
| Macleod et al. ^23^ | 2017 | Uses Levitas (2007) for operational definition | 1. Service provision and access 2. Civic participation 3. Social relations and resources |
| Madanipour ^31^ | 1998; 2015 | Social exclusion ‘is a societal, that is a society- wide, process, induced by wider changes and working itself through in specific ways shaped by national contexts and negatively affecting the ability of particular groups to participate in those social relationships which mean that ‘living in a place’ contributes to human flourishing’ (pg. 17) | 1. Economic 2. Political 3. Cultural |
| Nicholson ^32^ & Brothers of St. Lawrence ^33^ | 2008; 2010 | A social inclusion approach involves the building of personal capacities and material resources, to fulfil one’s potential for economic and social participation, and thereby a life of common dignity. | 1. Personal capabilities – health, education, social networks 2. Material resources – housing, transport, income, access to services 3. Employment - economic participation 4. Social participation – social connection, community |
| Pierson ^34^ | 2002 | “Social exclusion is a process that deprives individuals and families, groups and neighbourhoods of the resources required for participation in the social, economic and political activity of society as a whole.” |  |
| ﻿ Scharf et al. ^35^ | 2004 | Uses definitions from the Social exclusion unit (2001) and Berghman (1997). | 1. Exclusion from material resources 2. Exclusion from social relations 3. Exclusion from civic activities 4. Exclusion from basic services 5. Neighbourhood exclusion |
| Scutella et al ^4^ | 2012 |  | 1. Material/economic resources 2. Economic participation 3. Education/knowledge 4. Health and wellbeing 5. Social relations/participation 6. Political or community participation 7. Living environment 8. Access to services 9. Personal safety |
| Social Exclusion Unit (UK) ^18^ | 1997 | Social exclusion is a shorthand label for what can happen when people or areas suffer from a combination of linked problems such as unemployment, poor skills, low incomes, poor housing, high crime environments, bad health, and family breakdown. |  |
| Sommerville ^36^ | 1998 | Concepts of social exclusion are socially constructed by different combinations of economic, social, and political processes. It is suggested that the core meaning of social exclusion is bound up with social isolation and social segregation, and it is therefore argued that an analysis of social mobility (or the lack of it) is crucial to understanding the content and extent of social exclusion. | 1. Economic 2. Political 3. Moral |
| Tsakloglou & Papadopoulos ^37^ | 2001 | Key elements of social exclusion: *Multidimensional*; *Dynamic* (people are not just excluded because of their current situation but also because they have little prospect for the future); *Recognises agency* (lies beyond the narrow responsibility of the individual); *Relational* (there is discontinuity in the relationship of the individual with the rest of society) | 1. Income 2. Living conditions 3. Necessities of life 4. Social relations   Also measured time spent experiencing exclusion to capture dynamic nature. |
| Van Bergen et al. ^14^ | 2017 | Social exclusion “refers to the inability of certain groups or individuals to participate fully in society due to personal and societal factors. SE is a multidimensional concept, involving cumulative disadvantages.” | 1. Social - Limited social participation 2. Economic - Material deprivation 3. Political - Inadequate access to basic social rights 4. Lack of normative integration |

**Supplement B Variables included in the social exclusion score by domain**

We reviewed measures included in Wave 1 of *Understanding Society* and identified 28 variables which were related to the five domains of social exclusion.

**Supplemental table 2 Measures included in each domain**

| **Material exclusion (11 measures)** | **Relational exclusion**  **(6 measures)** | **Political exclusion**  **(7 measures)** | **Digital exclusion**  **(2-3 measures)** | **Structural exclusion**  **(4 measures)** |
| --- | --- | --- | --- | --- |
| - Household income - Educational attainment - Income satisfaction - Subjective financial situation - Falling behind on bills - Unable to afford material goods - Employment status - Job satisfaction - Would like to move house - Housing tenure - Housing affordability (<30% of income) | - Marital status - Household composition - Relationship satisfaction and cohesion (Wave 1 only) - Loneliness and social isolation (Wave 10 only) - Contact with extended family - Neighbourhood cohesion - Local friends mean a lot | - Supports a political party - Level of interest in politics - Satisfaction with environmental habits - Environmental lifestyle - Individual behaviours contribute to climate change - Worth taking individual action - Attends religious services | - Home computer and internet access - Mobile phone in household - Frequency of internet use (Wave 10 only) | - Educational mobility - Occupational mobility - Difficulties with English - Regional inequalities (GINI index) |

**Supplement C – Principal component analysis result (Wave 1, 2009/10)**

*Material exclusion*

Figure 1 Material exclusion – Scree plot

Table 1 Material exclusion – variance explained by components

| Component | Eigenvalue | Difference | Proportion | Cumulative |
| --- | --- | --- | --- | --- |
|  |  |  |  |  |
| Comp1 | 3.15 | 1.30 | 0.29 | 0.29 |
| Comp2 | 1.85 | 0.82 | 0.17 | 0.45 |
| Comp3 | 1.03 | 0.09 | 0.09 | 0.55 |
| Comp4 | 0.94 | 0.07 | 0.09 | 0.63 |
| Comp5 | 0.87 | 0.06 | 0.08 | 0.71 |
| Comp6 | 0.80 | 0.20 | 0.07 | 0.78 |
| Comp7 | 0.61 | 0.08 | 0.06 | 0.84 |
| Comp8 | 0.53 | 0.01 | 0.05 | 0.89 |
| Comp9 | 0.52 | 0.12 | 0.05 | 0.94 |
| Comp10 | 0.40 | 0.10 | 0.04 | 0.97 |
| Comp11 | 0.30 | . | 0.03 | 1.00 |

Table 2 Material exclusion - principal component loadings (unrotated)

|  | Comp1 | Comp2 | Comp3 | Comp4 | Comp5 | Unexplained variance |
| --- | --- | --- | --- | --- | --- | --- |
|  |  |  |  |  |  |  |
| Educational attainment | 0.19 | -0.25 | 0.38 | -0.38 | 0.71 | 0.06 |
| Income fifths | 0.37 | -0.23 | -0.01 | -0.06 | 0.18 | 0.43 |
| Income satisfaction | 0.33 | 0.18 | 0.40 | 0.01 | -0.33 | 0.34 |
| Subjective financial status | 0.40 | 0.18 | 0.28 | -0.05 | -0.27 | 0.29 |
| Falling behind on bills | 0.29 | 0.18 | -0.08 | -0.18 | -0.04 | 0.63 |
| Unable to afford material goods | -0.43 | -0.14 | -0.06 | 0.07 | 0.10 | 0.35 |
| Housing affordability (<30%) | 0.26 | 0.25 | -0.56 | -0.05 | 0.15 | 0.33 |
| Housing tenure | 0.33 | 0.10 | -0.47 | 0.07 | 0.20 | 0.37 |
| Would like to move house | 0.10 | 0.24 | 0.23 | 0.84 | 0.39 | 0.02 |
| Employment status | 0.26 | -0.54 | -0.05 | 0.22 | -0.17 | 0.18 |
| Job satisfaction | -0.16 | 0.59 | 0.14 | -0.23 | 0.17 | 0.18 |

Table 3 Material exclusion - principal component loadings (Promax rotation)

|  | Comp1 | Comp2 | Comp3 | Comp4 | Comp5 | Unexplained variance |
| --- | --- | --- | --- | --- | --- | --- |
|  |  |  |  |  |  |  |
| Educational attainment |  |  |  | 0.95 |  | 0.06 |
| Income fifths |  | -0.25 |  | 0.30 |  | 0.43 |
| Income satisfaction | 0.63 |  | -0.20 |  |  | 0.34 |
| Subjective financial status | 0.59 |  |  |  |  | 0.29 |
| Falling behind on bills | 0.24 |  | 0.24 |  |  | 0.63 |
| Unable to afford material goods | -0.42 |  |  |  |  | 0.35 |
| Housing affordability (<30%) |  |  | 0.69 |  |  | 0.33 |
| Housing tenure |  |  | 0.62 |  |  | 0.37 |
| Would like to move house |  |  |  |  | 0.99 | 0.02 |
| Employment status |  | -0.66 |  |  |  | 0.18 |
| Job satisfaction |  | 0.69 |  |  |  | 0.18 |

*Relational exclusion*

*Figure 2 Relational exclusion scree plot*

**

Table 4 Relational exclusion – variance explained by components

| Component | Eigenvalue | Difference | Proportion | Cumulative |
| --- | --- | --- | --- | --- |
| Comp1 | 2.34 | 0.68 | 0.39 | 0.39 |
| Comp2 | 1.66 | 0.65 | 0.28 | 0.67 |
| Comp3 | 1.01 | 0.44 | 0.17 | 0.84 |
| Comp4 | 0.57 | 0.31 | 0.09 | 0.93 |
| Comp5 | 0.26 | 0.11 | 0.04 | 0.98 |
| Comp6 | 0.15 | . | 0.02 | 1.00 |

Table 5 Relational exclusion - principal component loadings (unrotated)

|  | Comp1 | Comp2 | Comp3 | Unexplained variance |
| --- | --- | --- | --- | --- |
|  |  |  |  |  |
| Household composition | 0.58 | -0.17 | 0.07 | 0.16 |
| Marital status | 0.58 | -0.22 | -0.03 | 0.14 |
| Relationship satisfaction + cohesion | 0.20 | 0.01 | 0.90 | 0.09 |
| Frequency of family contact | 0.24 | 0.66 | -0.10 | 0.13 |
| Importance of local friendships | 0.28 | 0.64 | -0.08 | 0.13 |
| Neighbourhood cohesion | 0.39 | -0.29 | -0.41 | 0.34 |

Table 6 Relational exclusion - principal component loadings (promax rotated, <0.2 excluded)

|  | Comp1 | Comp2 | Comp3 | Unexplained variance |
| --- | --- | --- | --- | --- |
|  |  |  |  |  |
| Household composition | 0.58 |  |  | 0.16 |
| Marital status | 0.61 |  |  | 0.14 |
| Relationship satisfaction + cohesion |  |  | 0.92 | 0.09 |
| Frequency of family contact |  | 0.71 |  | 0.13 |
| Importance of local friendships |  | 0.70 |  | 0.13 |
| Neighbourhood cohesion | 0.53 |  | -0.33 | 0.34 |

*Political exclusion*

*Figure 3 Political exclusion scree plot*

**

Table 7 Political exclusion – variance explained by components

| Component | Eigenvalue | Difference | Proportion | Cumulative |
| --- | --- | --- | --- | --- |
| Comp1 | 1.55 | 0.40 | 0.22 | 0.22 |
| Comp2 | 1.15 | 0.12 | 0.16 | 0.39 |
| Comp3 | 1.03 | 0.02 | 0.15 | 0.53 |
| Comp4 | 1.00 | 0.11 | 0.14 | 0.68 |
| Comp5 | 0.89 | 0.10 | 0.13 | 0.80 |
| Comp6 | 0.80 | 0.22 | 0.11 | 0.92 |
| Comp7 | 0.58 | . | 0.08 | 1.00 |

Table 8 Political exclusion – principal component loading (unrotated)

|  | Comp1 | Comp2 | Comp3 | Comp4 | Unexplained variance |
| --- | --- | --- | --- | --- | --- |
|  |  |  |  |  |  |
| Supports a political party | 0.55 | 0.43 | -0.17 | 0.10 | 0.28 |
| Level of interest in politics | 0.61 | 0.24 | -0.20 | 0.01 | 0.31 |
| Satisfied with environmental habits | -0.20 | 0.36 | 0.26 | 0.71 | 0.21 |
| Environmental lifestyle | 0.35 | -0.27 | 0.48 | 0.40 | 0.33 |
| Individual behaviours contribute to climate change | -0.13 | 0.42 | 0.59 | -0.22 | 0.37 |
| Worth taking individual action | -0.26 | 0.61 | -0.14 | -0.18 | 0.41 |
| Attends religious services | 0.27 | -0.01 | 0.51 | -0.50 | 0.37 |

Table 9 Political exclusion – principal component loading (promax rotation)

|  | Comp1 | Comp2 | Comp3 | Comp4 | Unexplained variance |
| --- | --- | --- | --- | --- | --- |
|  |  |  |  |  |  |
| Supports a political party | 0.72 |  |  |  | 0.28 |
| Level of interest in politics | 0.68 |  |  |  | 0.31 |
| Satisfied with environmental habits |  |  | 0.87 |  | 0.21 |
| Environmental lifestyle |  | 0.73 | 0.27 |  | 0.33 |
| Individual behaviours contribute to climate change |  |  |  | 0.72 | 0.37 |
| Worth taking individual action |  | -0.65 |  |  | 0.41 |
| Attends religious services |  |  | -0.34 | 0.67 | 0.37 |

*Digital exclusion*

*Figure 4 Digital exclusion scree plot*

**

Table 10 Digital exclusion – variance explained by components

| Component | Eigenvalue | Difference | Proportion | Cumulative |
| --- | --- | --- | --- | --- |
|  |  |  |  |  |
| Comp1 | 1.96 | 0.78 | 0.33 | 0.33 |
| Comp2 | 1.18 | 0.20 | 0.20 | 0.52 |

Table 11 Digital exclusion – Principal component loadings (unrotated)

Comp1 Comp2

Computer + internet -.7071 .7071

Mobile phone .7071 .7071

*Structural exclusion*

*Figure 5 Structural exclusion scree plot*

**

Table 12 Structural exclusion – variance explained by components

| Component | Eigenvalue | Difference | Proportion | Cumulative |
| --- | --- | --- | --- | --- |
|  |  |  |  |  |
| Comp1 | 1.96 | 0.78 | 0.33 | 0.33 |
| Comp2 | 1.18 | 0.20 | 0.20 | 0.52 |
| Comp3 | 0.98 | 0.16 | 0.16 | 0.68 |
| Comp4 | 0.82 | 0.05 | 0.14 | 0.82 |
| Comp5 | 0.77 | 0.46 | 0.13 | 0.95 |
| Comp6 | 0.31 | . | 0.05 | 1.00 |

Table 13 Structural exclusion – principal component loading (unrotated)

|  | Comp1 | Comp2 | Comp3 | Unexplained |
| --- | --- | --- | --- | --- |
| Difficulty with day-to-day English | 0.21 | -0.21 | 0.89 | 0.08 |
| Intergenerational educational mobility | -0.12 | 0.67 | 0.36 | 0.32 |
| Intergenerational occupational mobility | -0.23 | 0.64 | 0.03 | 0.42 |
| Regional Gini (wealth inequality) | 0.41 | 0.02 | 0.08 | 0.66 |

Table 14 Structural exclusion – principal component loading (promax rotation)

|  | Comp1 | Comp2 | Comp3 | Unexplained |
| --- | --- | --- | --- | --- |
| Difficulty with day-to-day English |  |  | 0.95 | 0.08 |
| Intergenerational educational mobility |  | 0.76 |  | 0.32 |
| Intergenerational occupational mobility |  | 0.65 |  | 0.42 |
| Regional Gini (wealth inequality) | 0.37 |  |  | 0.66 |

*Figure Overall PCA – Scree plot*

**

*Overall PCA of all variables to confirm clustering by domain*

We could explain 40.0% of the variance by retaining six components. Material exclusion variables strongly loaded on two components, and we retained six components. Material exclusion variables loaded onto Component 1 (financial) and 2 (education and employment). Component 3 included relational variables including neighbourhood relationships and extended family contact, Component 4 included political exclusion variables including strong loading for political interest and environmental lifestyle variables. Component 5 included digital exclusion variables, but also included household relationship variables from the relational exclusion domain. Finally, Component 6 included strong loading for intergenerational mobility and regional inequality. This overall PCA confirms the high correlation between variables included in each domain, providing additional justification for their assignment. Retaining six components explained 40.0% of the variance, so to reach the pre-specified cut-off of 65% variance, we needed to retain 14 components. There was still broad grouping by domain, but with multiple components for each domain.

**Supplement D – Principal component analysis result (Wave 10, 2018/19)**

*Material exclusion*

Figure 1 Material exclusion – Scree plot

**

*Figure 2 Relational exclusion scree plot*

**

*Figure 3 Political exclusion scree plot*

**

*Figure 4 Digital exclusion scree plot*

**

*Figure 5 Structural exclusion scree plot*

**
